# Access and utilisation of maternity care for disabled women who experience domestic abuse: a systematic review

**DOI:** 10.1186/1471-2393-14-234

**Published:** 2014-07-17

**Authors:** Jenna P Breckenridge, John Devaney, Thilo Kroll, Anne Lazenbatt, Julie Taylor, Caroline Bradbury-Jones

**Affiliations:** 1 Social Dimensions of Health Institute, University of Dundee, Dundee, UK; 2 School of Sociology, Social Policy and Social Work, Queen’s University Belfast, Belfast, UK; 3 Child Protection Research Unit, NSPCC/University of Edinburgh, Edinburgh, UK; 4 School of Nursing, Midwifery and Social Work, University of Manchester, Manchester, UK

**Keywords:** Disability, Domestic abuse, Pregnancy, Maternity, Access, Utilisation, Review

## Abstract

**Background:**

Although disabled women are significantly more likely to experience domestic abuse during pregnancy than non-disabled women, very little is known about how maternity care access and utilisation is affected by the co-existence of disability and domestic abuse. This systematic review of the literature explored how domestic abuse impacts upon disabled women’s access to maternity services.

**Methods:**

Eleven articles were identified through a search of six electronic databases and data were analysed to identify: the factors that facilitate or compromise access to care; the consequences of inadequate care for pregnant women’s health and wellbeing; and the effectiveness of existing strategies for improvement.

**Results:**

Findings indicate that a mental health diagnosis, poor relationships with health professionals and environmental barriers can compromise women’s utilisation of maternity services. Domestic abuse can both compromise, and catalyse, access to services and social support is a positive factor when accessing care. Delayed and inadequate care has adverse effects on women’s physical and psychological health, however further research is required to fully explore the nature and extent of these consequences. Only one study identified strategies currently being used to improve access to services for disabled women experiencing abuse.

**Conclusions:**

Based upon the barriers and facilitators identified within the review, we suggest that future strategies for improvement should focus on: understanding women’s reasons for accessing care; fostering positive relationships; being women-centred; promoting environmental accessibility; and improving the strength of the evidence base.

## Background

Domestic abuse during pregnancy has such negative consequences for maternal and infant health that the World Health Organization (WHO) has declared it a significant global concern [1]. More than 30% of domestic abuse begins during pregnancy [2,3] and evidence suggests that pre-existing abuse may escalate during the prenatal period [4-6]. Although 10% of women giving birth in the United Kingdom (UK) are reported to have some degree of disability, there is little understanding of disabled women’s experiences of domestic abuse during pregnancy. Disabled women are two times more likely to suffer physical abuse from an intimate partner than non-disabled women [7], and it is therefore likely that disabled women may be particularly vulnerable to pregnancy-related abuse. Nixon [8] has suggested that disabled women who experience domestic abuse face compound oppressions. Several studies have linked domestic abuse with adverse maternal and infant outcomes [9-13]. Potentially compounding these negative consequences, certain disabled women may be more susceptible to pregnancy complications than non-disabled women [14,15]. Moreover, studies have suggested that abused women delay accessing maternity services until the third trimester [16-18] and that disabled women are also likely to have delayed or suboptimal access to healthcare [14,19,20].

Disability and domestic abuse during pregnancy may therefore have compounding effects on women’s access to and utilisation of maternity services, placing them at increased risk of undetected pregnancy complications. As yet, however, there is little understanding of the relationship between disability, domestic abuse and access to maternity care. Previous research in the UK [21,22] and the United States (USA) [23,24] has provided some insight into disability and domestic abuse more generally, however little is known about how domestic abuse impacts upon disabled women’s access to and use of maternity care. Until there is a good understanding of the factors that compromise or facilitate disabled women’s access and utilisation of maternity services when they experience domestic abuse, the priority areas for improving access and utilisation remain elusive.

The purpose of this systematic review was to explore the antecedents and consequences of inadequate access to maternity care when disability and domestic abuse co-exist. By summarising and synthesising the literature relating to disability, domestic abuse and access to maternity care, the review supports future development of robust improvement strategies and provides direction for future research.

## Methods

Although typically associated with reviews of randomised controlled trials, it is now recognised that the standard approach to systematic reviews can be adopted for different questions and study designs [25]. Our systematic review addressed the following questions in relation to disabled women experiencing domestic abuse:

1. What are the barriers that compromise access to and utilisation of maternity services?

2. What are the facilitators to accessing and utilising such services?

3. What are the consequences of inappropriate and/or delayed access to maternity care for women’s reproductive health and wellbeing?

4. How effective are existing strategies to enhance access and utilisation of maternity services?

### Key definitions

Domestic abuse, also referred to as domestic violence, intimate partner violence or violence against women, is defined by WHO as “physical, sexual or mental harm or suffering… including threats of such acts, coercion or arbitrary deprivation of liberty, whether occurring in public or in private life” [26]. This systematic review forms part of a larger study of the relationship between domestic abuse, disability and access to maternity care in the UK and therefore, for the purposes of the review, the WHO definition is supplemented by the UK policy definition of domestic abuse: “any incident or pattern of incidents of controlling, coercive or threatening behaviour, violence or abuse between those aged 16 or over who are or have been intimate partners or family members regardless of gender or sexuality” [27]. This includes psychological, physical, sexual, financial and emotional abuse. Generally within the UK, the term ‘abuse’ is preferred over ‘violence’ because this most adequately captures the range of abusive behaviours extending beyond physical abuse.

We used the term ‘disabled’ as defined by the United Nations to refer to any person with “long-term physical, mental, intellectual or sensory impairments which in interaction with various barriers may hinder their full and effective participation in society on an equal basis with others” [28]. This definition is supplemented by the UK Government Equality Act [29], where ‘long term’ refers to a health condition or impairment which lasts longer than 12 months, or is likely to reoccur within 12 months. The term ‘disabled women’ is preferred to ‘women with disabilities’ as this reflects the social model of disability, which contends that people have impairments but are disabled by social factors [21]. The definitions of disability and domestic abuse were intentionally broad in order to increase the sensitivity of the literature search and ensure that there were a sufficient number of articles to review. ‘Maternity care’ relates to maternity care of any kind, including primary and/or secondary care, pre and post-natal care, and private, voluntary or state funded services. ‘Access’ to services is defined as having the opportunity to use maternity services, whilst ‘utilisation’ refers to the actual or realised use of services [30].

### Search strategy

A systematic approach was used to minimise bias and reduce the risk of errors or omissions [31]. To access data about the health, social and psychological dimensions of the review questions, six electronic databases were searched, encompassing literature from 1946 to 2013 (Medline, Embase, Cinahl, ASSIA, SSCI, and PsycINFO). This time-frame ensured that the search was comprehensive and would capture all relevant papers. For pragmatic reasons, the search was limited to English language titles. No other limits or filters were applied. It was anticipated that studies may be indexed under either ‘disability’ or ‘domestic abuse’ and so, to avoid missing relevant data, ‘maternity’ and ‘disability, and ‘maternity’ and ‘domestic abuse’ were searched separately before combining the results. Table 1 summarises the basic search strategy. Search strings were created in each category, using a combination of subject headings (e.g. MeSH) and key words. Multiple synonyms and related terms were used e.g. ‘domestic violence’, ‘intimate partner violence’ etc. These are demonstrated in Additional file 1, which shows the detailed search process used in Medline and Embase.

**Table 1:** Basic search strategy

1.	Maternity
2.	Disability
3.	Domestic abuse
4	1 and 2
5.	1 and 3
6.	4 or 5

The electronic database search yielded 6007 potentially relevant articles. A hand search of journals in the field yielded a further 162 potentially relevant articles. A total of 6169 abstracts were therefore screened for inclusion. All titles and abstracts were screened against the inclusion criteria by four pairs of independent reviewers (n = 8). Each pair screened 1000-1800 abstracts and, although time consuming, this made the process manageable. To ensure adherence to the protocol, one member of the research team (JPB) took responsibility for co-ordinating the screening process. Abstracts were included for review on the basis of the inclusion and exclusion criteria presented in Table 2. If it was unclear from the abstract whether or not a paper met all four inclusion criteria, it was taken forward to the next stage of screening.

**Table 2:** Inclusion and exclusion criteria

Inclusion:	Presents empirical data (either qualitative or quantitative)
	Focuses on or includes maternity care access and utilisation
	Focuses on or includes disabled women
	Focuses on or includes domestic abuse
Exclusion:	No empirical data presented
	Does not focus on access and utilisation of maternity or related primary care services
	Focuses on men only
	Focuses solely on child abuse (under 16 years), elder abuse, abuse by formal carers or abuse that occurred outside a pre-existing intimate or familial relationship
	Focuses solely on pregnancy outcomes and complications that are not associated with domestic abuse or issues of access and utilisation

### Selection

**Figure 1:**
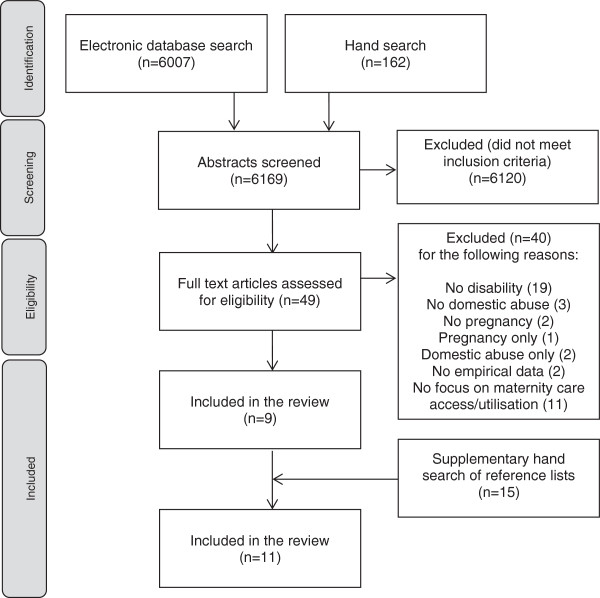
Flow diagram of the screening process.

Forty-nine full text articles were screened for eligibility against the inclusion criteria. All articles were read in full by the first author and then reviewed independently by other members of the team to moderate the screening process (each member of the team read 7 full text articles). As recommended by the Preferred Reporting Items for Systematic Reviews and Meta-Analyses (PRISMA) statement (http://www.prisma-statement.org), Figure 1 provides a flow diagram of the full screening process. Nine papers met all four inclusion criteria and were included for review. Although a total of twenty studies included all three key elements (disability, domestic abuse and pregnancy), only nine focused upon access and utilisation of maternity services. Figure 1 documents the reasons for exclusion of the remaining articles. To ensure an exhaustive search and prevent omissions, Barroso and colleagues [32] have recommended that researchers continually evolve their search strategy. A final hand search was therefore conducted using the reference lists of the nine included papers. This yielded a further 15 papers of interest, two of which met all four inclusion criteria after independent review by two authors.

### Data extraction and analysis

A standard form was designed to structure the data extraction process, using the headings: setting; aims; sample; methods; findings; relevance to review questions; and methodological critique. The first author extracted data from each of the included studies and tabulated the findings under each heading. Data extraction was double checked by the rest of the research team and any disagreements were resolved through discussion. Methodological critique was supported by reference to the Critical Appraisal Skills Programme (CASP) checklists for observational studies (http://www.casp-uk.net). Given that qualitative research is distinctly different to quantitative research, a different framework was used to support critical appraisal of the qualitative studies included within the review [33]. The rigid use of checklists has been criticised within qualitative research and Barbour [34] has argued that appraisal checklists should be used flexibly and in a manner that is apposite to individual study design. Although we were guided by Walsh and Downe’s [33] criteria for assessing qualitative studies, we were more concerned with a global assessment of quality rather than firm adherence to the checklist. The studies were of varied design and quality, however all were included in order to capture the broad range of perspectives in this area and permit reflection on the current quality of the evidence base. This is addressed later in the paper. Data from all eleven included studies were synthesised by categorising them under the four review questions. Data relating to each of the review questions were analysed inductively to identify themes and make comparisons across studies.

## Results

Eleven articles met the inclusion criteria and are summarised in a table (Additional file 2). As indicated in the table, the majority of studies were conducted in the USA (n = 6). Two studies were conducted in Brazil, one in India, one in Zambia and one in Australia. Eight studies used quantitative methods, one study used a qualitative approach and two studies utilised both quantitative and qualitative data. Five studies surveyed pregnant women [35-39], three utilised a prospective cohort design [40-42] and one tracked pregnant women through police records [43]. One study sought the views of health professionals only [44], whilst another interviewed both women and practitioners [45]. By identifying barriers and facilitators to accessing and using maternity care, the majority of studies addressed review questions one and two, with fewer data to support questions three and four. The results are expounded below.

### Barriers that compromise access and utilisation of maternity care

Eight studies highlighted barriers which compromise access to maternity care [35,37,38,40-42,44,45], relating to: mental health diagnosis; poor relationships with health professionals; environmental barriers; domestic abuse.

### Mental health diagnosis

Three studies hypothesised that mental illness is linked to inadequate maternity care [35,40,41]. In Ferri et al’s [35] study of the interactive effects of violence and mental disorder on maternal health, nearly 30% of their sample (n = 930) received less than the recommended six antenatal appointments. For women with a common mental health disorder (n = 226, defined as depression, anxiety, post-traumatic stress disorder, somatoform or dissociative disorder) 25.1% had between one and five antenatal appointments and 14.3% received no antenatal care at all. Interestingly, however, Ferri et al [35] identified similar statistics for women without a mental health disorder who experienced domestic abuse. They have suggested that mental illness and domestic abuse have independent, rather than compounding, effects on access to services. This is similar to the study by Huth-Bocks et al. [40] which reported that, although maternal depression was significantly associated with domestic abuse, it did not account for abused women’s later entry into prenatal care. Thus, whilst there is evidence to suggest that a mental health diagnosis can compromise access and utilisation of maternity services for women experiencing domestic abuse, the exact nature of this relationship is unclear.

Kim et al. [41] found that current psychiatric diagnosis had no adverse effect on the frequency and timing of antenatal visits; however, they also identified that women with a past psychiatric illness were significantly likely to be non-compliant with at least 50% of their scheduled antenatal appointments. This suggests that longer term conditions may present women with greater difficulties in accessing care. Having assessed psychiatric symptoms at single time points during women’s pregnancies, Ferri et al. [35] and Huth-Bocks et al. [40] were not able to account for the effects of long term mental health issues. Thus, a full understanding of the impact of a mental health condition on maternity care access and utilisation is difficult to ascertain.

### Poor relationships with health professionals

Three studies highlighted that negative past healthcare experiences, poor relationships with health professionals and fear or judgement from staff could compromise women’s access to services [37,44,45]. In Nosek et al’s [37] survey of women with physical impairments (n = 475), 26% of women lacked confidence in their care provider, believing that their physician was ill-informed about the impact of their disability on reproductive health. This lack of knowledge often manifested in women being refused treatment: 31% of participants in Nosek et al’s [37] study were refused care because of their disability and both Kopac and Fritz [44] and Smith et al. [45] noted that maternity care providers were reticent to provide treatment to ‘high risk’ women. Many disabled women feared that practitioners would condemn their pregnancies as abnormal, dangerous or wrong [45], with many being advised against pregnancy [37]. Both Kopac and Fritz [44] and Nosek et al. [37] identified that ineffective communication between staff and patients prevented women from getting appropriate reproductive healthcare. Factors influencing poor communication include: professionals’ lack of patience; lack of empathy; and a limited knowledge and understanding of disability issues [44]. Moreover, although very few women in Smith et al’s [45] study of disabled women’s access to maternity care in Zambia actually reported negative experiences with staff, the anticipation in itself was enough to deter women from utilising services.

### Environmental barriers

The physical, geographical and institutional environments in which maternity care occurs can present several barriers to accessing and utilising services. Four studies suggested that maternity care facilities are ill-equipped to provide services for disabled women who experience domestic abuse [37,38,44,45]. In a study of 120 pregnant women in the USA with spinal cord injury, 56% reported that their local hospital could not accommodate their disability needs when they gave birth [37]. Similar findings emerged in a large nationally representative survey of pregnant women (n = 35,248) across India [38]. Of the women experiencing pregnancy related blindness (12%), nearly 60% reported that they were concerned about the quality of maternity services. This presents a significant organisational barrier to accessing care. In Kopac and Fritz’s [44] survey of nurses working in hospitals, community services and physician’s offices across the USA (n = 727), 65.5% stated that there was no one in their setting who specialised in working with disabled women (specifically women with intellectual disabilities) and 70% did not have the opportunity to undergo generic disability training within their organisation. Many services therefore lack the staffing resources to meet the needs of disabled women.

Organisational and financing policy may also restrict disabled women’s access to care. According to Kopac and Fritz [44], many services choose not to treat women insured through Medicaid or Medicare (the social insurance systems in the USA that support disabled people or those on low income). There may also be restrictions within these policies themselves, whereby insurance schemes will not fund certain procedures or cater for the extra time required to carry out examinations when accommodating women with additional needs. Moreover, in countries where social insurance systems do not exist, the financial barriers to accessing maternity care are great, particularly for disabled women [38]. The high cost of transport was highlighted in two studies [38,45] and this was further compounded when women were refused treatment in their local hospital because of their disability and had to find care elsewhere. Public transport was also often inaccessible for women with mobility issues, adding to existing barriers to care.

Physical inaccessibility is a major barrier to the effective utilisation of maternity services and all four studies identified problems with the physical environment [37,38,44,45]. 7% of nurse respondents in Kopac and Fritz’s [44] survey (n = 727) found it difficult to arrange examinations for disabled women as a result of inaccessible offices, improper examination tables and inadequate equipment. Speaking to Nosek et al. [37] about her experience of maternity care, one woman was shocked that practitioners were not monitoring her weight: “could you believe that all through my pregnancy … they don’t know how much weight I’ve gained, because they don’t have a wheelchair or sitting scale” (p.22). Unlike Nosek et al’s study [37], Kopac and Fritz’s [44] findings are based only on the experiences of healthcare providers rather than disabled service users. It is thus possible that problems with physical accessibility are more significant to women than practitioners perceive. Over 26% of the nurses sampled did not respond to the question about barriers to accessing services, perhaps cementing the argument that practitioners may lack knowledge about the unique needs of disabled women.

### Domestic abuse

Nunes et al. [42], Huth-Bocks et al. [40] and Kim et al. [41] all concluded that domestic abuse is significantly associated with delayed entry into antenatal care for women with and without a mental health condition. It could therefore be suggested cautiously that domestic abuse and mental illness have independent effects on service access and utilisation. For women with a physical health condition, however, physical barriers to care can be amplified in the presence of domestic abuse, particularly when women are reliant on their partners for physical assistance and transport to appointments. Many women in Nosek et al’s [37] survey reported that their partner had removed mobility devices, withheld transportation or refused personal care. In Pandey et al’s [38] study of pregnancy related blindness in India, blind women were significantly more likely than women without blindness to have controlling husbands and limited autonomy to make decisions about their own health. Conversely, women who were empowered to make their own decisions had more positive health outcomes [38]. For women with physical and sensory impairments, then, the effects of domestic abuse may compound existing barriers to their access and utilisation of maternity care.

### Factors that facilitate access and utilisation of maternity care

Six studies identified enabling factors that could facilitate potential and realised access to maternity services [37-41,43]. Typically, the factors that facilitated access and utilisation of services were direct opposites of the barriers identified above e.g. good relationships with staff or physical accessibility. Two additional factors were identified as potentially increasing access and utilisation of services: 1. health needs arising from physical abuse; 2. support from friends and family.

### Health consequences of domestic abuse

Although domestic abuse has been identified as a barrier to accessing services, three studies identified that the health consequences of domestic abuse could actually prompt women to access services more quickly or utilise services more frequently [39,40,43]. Women experiencing physical violence during pregnancy were more likely than non-abused women to be hospitalised because of physical injuries [43]. Huth-Bocks et al. [40] reported that women experiencing both physical and emotional abuse had longer stays in hospital, visited the emergency room more frequently and had a higher number of visits to their doctor for the infant during the postnatal period than non-abused women. Similarly, in a study focusing predominantly on the effects of physical abuse on maternal and infant outcomes, Webster et al. [39] found that abused women had a significantly higher number of pregnancy-related hospital admissions than non-abused women.

It is suggested therefore that the consequences of domestic abuse on women’s physical health can amplify the need to utilise services during pregnancy. Even when women face barriers to care, such as the effects of a long term mental health condition, these may be overridden by immediate treatment needs which catalyse health service use. During pregnancy, women’s sense of necessity may be heightened and domestic abuse may cause women to worry more about the health of the baby than their own health and well-being [40]. ‘Necessity’ is a subjective concept and women will interpret and respond to their current health issues in different ways. Although women may be forced into accessing services because of immediate treatment needs [43], they may also make judgments about the importance of maternity care prior to accessing services. The impact of women’s decision making, and their actual and perceived need for treatment, are discussed later in the paper.

### Social support

It is well established in the general domestic abuse literature that social support facilitates maternity care access and utilisation. Huth-Bocks et al. [40] identified that, for women with mental health issues attending hospital and community based prenatal care (n = 202), social support moderated between severe domestic violence and negative maternal health outcomes. By facilitating earlier access to services, positive social relationships in turn resulted in improved health. Disabled women who experience domestic abuse, however, are likely to have small support networks, meaning that they miss out on social support as a protective factor [39]. Moreover, not all social relationships are supportive and women may fear the judgment of others. Smith et al. [45] reported that disabled women attending maternity clinics were subjected to gossip and stereotyping by other non-disabled women in the waiting room. Thus, whilst social support has the potential to facilitate access and utilisation of maternity services, this may not have been fully realised for disabled women.

### Consequences of delayed or inappropriate maternity care on women’s health and wellbeing

Physical and psychological consequences of inadequate care were documented equally within the review papers: three studies identified direct consequences for women’s physical health [35,38,42] and three studies reported on the emotional consequences of inadequate care [37-39]. In Pandey et al’s [38] study of pregnant women throughout India (n = 35,248), only 37% achieved the WHO recommended minimum of four prenatal visits. Even after controlling for other risk factors, women who were concerned about the distance, cost and quality of maternity services were significantly more likely to develop blindness during pregnancy than women with satisfactory access to care. Under-utilisation of maternity services has also been linked to insufficient pregnancy weight gain [42] and infants with low birth weight [35]. Both Nunes et al. [42] and Ferri et al. [35] focused predominantly on infant outcomes, giving only a limited insight into the direct consequences of inadequate care on maternal health. However, infant outcomes may be a telling reflection of maternal wellbeing. In relation to the emotional and psychological consequences of inadequate maternity care, Webster et al. [39] reported that women with fewer prenatal visits had more depressive symptoms than women who had adequate prenatal care. Women’s emotional wellbeing may also be compromised when they have limited involvement in making decisions about their own health [37,38].

Failure to recognise domestic abuse within maternity services was highlighted as risky to maternal and infant health and authors have raised concerns about the potentially negative consequences if domestic abuse is not sufficiently addressed [44,38]. While Mitra et al. [36] reported that practitioners were equally likely to ask disabled women about domestic abuse as non-disabled women, Kopac and Fritz [44] uncovered a lack of attention to disabled women’s experiences of domestic abuse within gynaecological and reproductive health services. The contrasting findings may be attributable to different samples within both studies: Kopac and Fritz [44] focused explicitly on women with developmental disabilities and may therefore have encountered more communication difficulties. Alternatively, Mitra et al’s [36] study is more recent and may reflect the greater awareness of domestic abuse within current policy and practice. Although they identified appropriate screening processes, Mitra et al. [36] were unable to ascertain whether disabled women received appropriate referrals to domestic abuse agencies following disclosure. This is an important consideration, given Nosek et al’s [37] finding that disabled women face serious barriers to accessing existing programs that help women remove violence from their lives. Without due consideration of the social factors influencing women’s health and wellbeing, inappropriate maternity care may be inconsequential or further compound negative health outcomes.

### Strategies for improving access and utilisation of maternity services for disabled women who experience domestic abuse

Only one study identified strategies used by maternity services to improve disabled women’s access to and utilisation of care. The safe motherhood and reproductive health services featured in Smith et al’s [45] study aimed to improve access for disabled women by minimising the effects of poverty and stigma. To make services more financially accessible, family planning, antenatal and postnatal care were provided free of charge. This did not address additional costs, however, such as prescription charges or the cost of transportation. Similarly, the authors concluded that, while attempts to tackle stigma may have been well meaning, they had limited effectiveness. To protect disabled women from gossip or being stared at by other patients, they were either referred to a hospital outside their own community or were treated quickly and discretely within local clinics. ‘Sheltering’ disabled women from stigma in this way, however, may serve only to reinforce negative stereotypes that pregnancy is abnormal for disabled women; entrenching rather than removing stigma as a barrier to accessing care. While Smith et al. [45] identified that many of the disabled women accessing these services had experienced abuse in the form of sexual exploitation, their study did not explore whether or not this had an effect on women’s access to care and how it was addressed by maternity care practitioners. The evidence behind strategies for supporting disabled women’s access to maternity care when they experience domestic abuse is therefore very limited.

## Discussion

This systematic review has shown that access to maternity care for disabled women experiencing domestic abuse is influenced by multiple factors, including mental health issues, the effects of domestic abuse, social and professional relationships and the environment in which services are delivered. These barriers are consistent with studies of domestic abuse and pregnancy [46-49], and disability and pregnancy [14,19], which have independently explored the reasons for delayed prenatal care in both groups of women. To the best of our knowledge, this is the only review to date that explores the antecedents and consequences of inadequate maternity care when disability and domestic abuse co-exist. The majority of studies included in the review focused upon the factors that compromise access, suggesting that more is known about why women do not access care than about the potential negative consequences of inadequate care or how to improve access and utilisation. A stark finding was that only one study documented strategies for overcoming barriers to accessing care. On the basis of the review findings, we suggest that future research, policy and practice give further consideration to: understanding women’s reasons for accessing care; fostering positive relationships as a means to accessing care; being women-centred; promoting environmental accessibility; improving the strength of the evidence base.

### Understanding women’s reasons for accessing care

Several factors impact upon women’s utilisation of maternity services and it is permissible to draw conclusions about women’s access to care based upon the presence of certain barriers in their lives. It is pertinent to remember, however, that each woman will respond to barriers in different ways. Fundamentally, individuals must recognise a need for healthcare before actually using services; they must deem “their problems to be of sufficient importance and magnitude to seek professional help” [30] p.3. Our review identified that domestic abuse can create or exacerbate an immediate health need which makes health service utilisation unavoidable, for example a physical injury requiring medical attention [43]. While this may create an opportunity for women to receive needed prenatal care, full and effective utilisation of services can only be realised if healthcare staff identify a pregnancy-related treatment need and respond with appropriate referrals. Moreover, by the time women access services out of necessity it may be too late to prevent negative consequences for maternal and infant health. Evidence also suggests that the majority of domestic abuse takes the form of psychological abuse, coercion and control [50] and therefore the consequences of abuse may not always demand immediate medical attention.

Even in the absence of biological imperative, women make judgments about the necessity of accessing routine services [30]. Our review found that the difficulties associated with travel and fear of negative attitudes from staff often outweighed the perceived benefits of attending antenatal appointments [37,38,44,45]. Finlayson and Downe [51], in a metasynthesis of studies exploring why women in general do not use antenatal services in low and middle income countries, also identified that women continually weigh up their own priorities and beliefs against the expectation that they utilise care. Andersen [30] has differentiated this from the ‘actual need’ discussed earlier and women’s ‘perceived need’ for service utilisation. As a social phenomenon, the ‘need’ to seek professional healthcare is subjective and will be rationalised or exaggerated by outside factors. For example, social support was identified as having a positive effect on access to maternity care for women with severe levels of abuse, but not for those with lower levels of abuse [40]. This is perhaps because, for women with high levels of abuse, friends and family may stress the potential for negative consequences and emphasise the importance of accessing care. Conversely, for women experiencing low levels of abuse, social support may be seen as a replacement for professional input and women’s perceived need for maternity services may be smaller. To ensure future strategies for improving access to maternity care are effective, further research is required to understand women’s decision making processes more fully, particularly in the context of disability and domestic abuse where autonomous decision making may be restricted.

### Fostering positive relationships as a means to accessing care

Relationships have a critical influence on women’s utilisation of maternity care [37-42,44,45]. Poor relationships with maternity care practitioners in the past deter women from utilising services again [37,44]. Even when women have had no previous negative experiences, the anticipation alone makes women reticent to attend appointments [45]. Although Finlayson and Downe’s [51] findings are similar to our review, they did not focus specifically on domestic abuse or disability. Therefore, where they reported that women were reluctant to seek professional help for what is considered to be a ‘normal life event’, our review showed that disabled women may often be told that their pregnancies are ‘abnormal’. The internalisation of stigma and societal misconceptions can have a considerable impact on women’s perceived need for care and their willingness to use services. Walsh-Gallagher and colleagues [52] have warned maternity care practitioners against classifying all disabled pregnant women as ‘high risk’. Instead, professionals must establish positive, non-judgmental relationships with women and in so doing, change women’s negative perceptions of maternity care which are often a barrier to seeking help.

The extent to which maternity care practitioners are aware of the complexities arising from the combination of disability and domestic abuse remains unclear. Two studies recommended that maternity staff should receive additional education [37,44] and seven studies suggested that practitioners should know how to identify and respond to domestic abuse [35,36,39,40,42,43,45]. The need for education and training is supported by other literature [48,49,53] and international policy and strategy documents [54]. According to WHO [26], current training interventions are targeted typically at the identification of domestic abuse, without adequate training in further care or how to change judgmental attitudes and cultural stereotypes. Effective prenatal care relies not only upon early access to services but also the continued utilisation of services. In the first appointment, practitioners have only a short time in which to develop a positive relationship with women and encourage them to return for follow-up appointments. Further research is required to develop effective staff training, potentially drawing upon the key principles underlying positive practitioner-patient relationships identified within the review: effective communication, non-judgmental attitudes and encouraging active involvement in the treatment process.

Studies have shown that social relationships can have a positive or negative effect on women’s decisions to utilise maternity care [38-42,45]. Social support can promote early and continued utilisation of services; however disabled women may lack strong support networks, particularly in the context of domestic abuse [55]. Fostering positive relationships within the community is therefore essential and improving access to maternity care cannot be achieved by addressing internal service barriers alone. Outward looking improvement strategies could capitalise on social support as a resource and involve colleagues in community education and health promotion. In an earlier study of access to gynaecological services for women with developmental disabilities, Kopac et al. [53] identified that support staff and formal carers have a key role in prompting women to attend services and accompanying them to appointments. Formal support is available within the community and by developing positive relationships with other services and ensuring that agencies are well informed about the importance of early prenatal care, improved access to maternity care may be achieved through multidisciplinary collaboration.

### Being women-centred

Delayed prenatal care and infrequent utilisation of maternity services have negative consequences for women’s physical and psychological health and wellbeing [35,37-39,42]. Optimal access to maternity care, however, extends beyond the timing and frequency of antenatal appointments. Services must also support women to make autonomous and informed choices about their maternity journeys [37,38]. Although WHO [26] promote women’s active involvement in their care, our review suggests that this is not being actualised for disabled women who experience domestic abuse. Good ‘access’ to maternity care must be both physical and cognitive [56]. While ‘physical’ access refers to women’s physical presence at appointments, ‘cognitive’ access implies that women have understood the information given and that her needs have been fully understood by the health practitioner. Even when physical barriers have been removed, women may still experience restricted access to services if they are not fully engaged in the process. Services must therefore be women-centred and based on sound communication [49]. Adequate access to maternity care relies upon the quantity *and* quality of service provision.

### Promoting environmental accessibility

For disabled women, physical access may be a significant issue in itself and several studies identified problems with environmental accessibility [37,38,44,45]. Improvement strategies must tackle the physical, geographical, social, financial, organisational and political barriers facing disabled women who experience domestic abuse. Recent guidelines [26] reflect the need to address these barriers, however further work is needed to develop operational improvement strategies. Care providers must have adequate facilities and equipment to support disabled women [26,37,44]. At an organisational level, policies should support access to maternity services for disabled women experiencing domestic abuse and should not stymie women’s opportunities for referrals to additional services [54]. Simply asking about domestic abuse does not necessarily create the opportunity for women to receive more effective care and practitioners must have the knowledge and resources to provide appropriate support [57]. In the UK, where this review was undertaken, the Royal College of Nursing [58] and Royal College of Midwives [59] have produced guidelines on pregnancy and disability which emphasise that health professionals should be aware of how a woman’s impairment will affect her pregnancy, and how the pregnancy might in turn affect her health. These guidelines do not, however, mention anything about how to support disabled women who experience domestic abuse during their pregnancy. Given that nearly 50% of disabled women giving birth in the UK experience domestic abuse [15], it is essential that policy and organisational guidelines support practitioners to improve accessibility and provide appropriate care.

External barriers to care, such as the cost of transport, the provision of social insurance and the economic climate, remain a bigger challenge and are generally outside the control of individual maternity services. While services themselves cannot necessarily remove all of these barriers, any strategies for improving access and utilisation must match the economic and cultural contexts in which people live. In addition to providing ‘core’ maternity services, the *Global Action Report on Preterm Birth*[54] has recommended that social and financial support be integrated within routine antenatal care. Service developments like this should be based on the best evidence and future research should be directed at identifying, honing and evaluating the most effective models of antenatal service delivery. The nature of the social and financial barriers facing women may be different in the context of both disability and domestic abuse, potentially influencing the nature and scope of subsequent interventions. Empirical research is therefore also needed to specifically identify the most effective ways of supporting disabled women to overcome environmental barriers to maternity care when they are compounded by the effects of abuse.

### Improving the strength of the evidence base

This review has provided some new insights into the complex relationship between disability, domestic abuse and access to maternity care, although empirical studies are lacking. To ensure that improvement strategies are effective, they must be rooted in a strong evidence base. Reflecting on the methodological strengths and shortcomings of the studies included in this review, we recommend that research regarding the effects of domestic abuse on disabled women’s access to maternity care should be more visible, more consistent and more methodologically varied.

#### Increased visibility

Empirical studies of the relationship between disability, domestic abuse and pregnancy are difficult to locate because the literature is compartmentalised. The studies either: investigate the consequences of domestic abuse during pregnancy; explore disabled women’s experiences of domestic abuse; or identify pregnancy risks for disabled women. Data about the relationship between disability, domestic abuse and maternity care is also ‘hidden’ within broader studies; only two studies included in the review referenced these three elements explicitly in their titles [35,36]. Instead, studies either focused predominantly on disability with a minimal focus on domestic abuse [37,38,41,44,45], or focused predominantly on domestic abuse with limited attention to disability [39,40,42,43]. As a result, narrow search strategies may miss critical findings when studies are indexed either under disability or domestic abuse. Furthermore, findings about disability, domestic abuse and access to maternity care may be incidental. For example, Webster et al. [39] intended to explore the effects of domestic abuse during pregnancy and also identified a high incidence of epilepsy and asthma within their sample, making their findings relevant to our review. Empirical studies which address this complex relationship explicitly are therefore essential to strengthening the evidence base and facilitating meaningful conclusions.

#### Increased consistency

Each study took different perspectives on ‘domestic abuse’, with some offering specific definitions differentiated by type and severity [39] and assessed by standardised domestic abuse measures [35,40,42]. Other studies used very broad definitions: Nosek et al. [37] did not specify different types of abuse although noted that abuse was predominantly perpetrated by a husband or intimate partner; Kopac and Fritz [44] did not differentiate between partner abuse and abuse by family members or strangers; Smith et al. [45] stated that women had experienced “sexual exploitation”; and Kim et al. [41] did not provide a definition or state how abuse was identified. These differences ultimately affect the quality of the studies and compromise the confidence with which conclusions can be drawn about the effects of domestic abuse on access to maternity care. Both Mitra et al. [36] and Lipsky et al. [43] focused only on physical abuse, although the police reported incidents featured in Lipsky et al. [43] may have been more severe than Mitra et al’s [36] study of mild to moderate abuse. Pandey et al. [38] asked women about humiliation, control and physical abuse, although their findings were hampered by missing data. Women were also asked about domestic abuse at different times and incidents of domestic abuse during pregnancy may have gone unreported. Moreover, six studies sampled women already attending maternity services, meaning that women with no access to services, who were perhaps affected most severely by the consequences of disability and domestic abuse, were not represented within these studies [35,39-42,45].

Disability was similarly represented inconsistently across all eleven studies. Samples were typically polarised between women with physical health conditions [37,39,45] and those with mental health issues [35,40-43]. Mitra et al. [36] asked participants to self-identify if they had “physical, mental, or emotional problems” (p.803) but did not differentiate between these disability categories in their analysis. With the exception of Kopac and Fritz [44] and Pandey et al. [38], women with sensory impairments or learning disabilities were under-represented in the review. This limits the transferability of the review findings to these groups. Other than one study which reported that abusive partners directly prevented women’s access to care [37], all of the studies that identified barriers to maternity care were typically focused on the effects of disability, rather than the effects of abuse. This perhaps indicates that disability-related access problems have a greater impact on women’s access to care than domestic abuse. The evidence is still very limited, however, and more research is needed to explore the *non*-disability barriers for disabled women, particularly the effects of abusive partner behaviour. Furthermore, ‘disabled women’ are not a homogenous group and future research should continue to differentiate between different types of disability to allow fuller understanding of women’s experiences.

#### Increased variation

The majority of studies used quantitative methods and while such approaches can indicate associations between disability status, domestic abuse and prenatal care utilisation, more qualitative research could explicate the complex nature of the barriers facing disabled women. When considering the interplay between disability and domestic abuse, the challenge for researchers is in disentangling cause and effect; it is difficult to differentiate the independent or compounding effects of disability and domestic abuse when they are complexly intertwined. It is important therefore that future research explores more closely how women are affected by impairment related barriers, barriers associated with domestic abuse, and how these impact upon one another. While the barriers facing women with physical impairments have been considered from a qualitative perspective, studies about how mental illness impacts on access to maternity care have all been quantitative. Qualitative research may reveal connections that have not become evident in quantitative data. Further quantitative research is also necessary, and in contrast to Nunes et al. [42], Huth-Bocks et al. [40] and Ferri et al. [35], studies should explore the effects of long term mental health conditions on access to maternity care when accompanied by domestic abuse.

### Limitations

This review was based on eleven studies of varying quality and the limitations of individual studies have been discussed. The studies originated in the USA, Australia, Brazil, Zambia and India, potentially limiting transferability of findings to other countries, including the UK where this review was undertaken. Service delivery in each of these countries occurs within different economic, cultural and political contexts, rendering meaningful comparison across studies more difficult. Similarly, it is difficult to make comparisons across studies which focus on different impairments; for example, women with a visual impairment may experience significantly different barriers to women with anxiety disorder. However, given the paucity of literature relating to disability, domestic abuse and access to maternity care, it would not have been feasible to narrow the focus to a specific type of impairment. Instead, this review lays the foundation for future research by highlighting some of the general barriers and facilitators associated with disabled women’s access to maternity care when they experience domestic abuse.

The review was conducted in accordance with PRISMA guidelines (see Additional file 3) and the search strategy employed was flexible and sensitive to finding ‘hidden’ data. The search was limited to English language papers for pragmatic reasons but, given the international spread of the included studies, it may have been prudent to include non-English language papers. This is recommended for future reviews on this topic. While review questions one and two were addressed fully, we found limited information about the consequences of inadequate maternity care and strategies for improving access to services. Including non-English language papers may have yielded more data to address questions three and four.

## Conclusions

While this review has gone some way to understanding how the coexistence of disability and domestic abuse might impact upon maternity care utilisation, there is still limited understanding of the antecedent factors that prevent disabled women from accessing maternity services because of abusive partner behaviour. The review confirms that disability and domestic abuse affect women’s access to maternity care, although methodological complexities make it difficult to draw conclusions about the extent to which these have a compounding effect. The timing and frequency of prenatal appointments is determined by personal, social, organisational and environmental factors. We have made recommendations relating to: understanding women’s reasons for accessing care; fostering positive relationships; being women-centred; promoting environmental accessibility; and improving the strength of the evidence base. In addition to exploring the antecedents and consequences of domestic abuse for disabled women, future research must now actively explore potential solutions and develop robust strategies for improving access and utilisation of maternity services for this group. Table 3 summarises the priorities for research, policy and practice.

**Table 3:** Future priorities for research, policy and practice

Research	Explore the negative consequences of delayed or inappropriate maternity care for disabled women who experience domestic abuse
	Understand women’s reasons for accessing maternity services and the factors that influence their decision making, particularly disability and domestic abuse
	Further explore the effects of long term mental health conditions on access to maternity care when accompanied by domestic abuse
	Explore maternity care practitioners’ understanding of disability and domestic abuse and evaluate the effectiveness of existing staff education
	Identify, develop and evaluate the most effective models of antenatal service delivery for disabled women who experience domestic abuse
	Studies which focus explicitly upon disability, domestic abuse and access to maternity care, including more qualitative research
Policy	Organisational policies and guidelines which account for the co-existence of disability and domestic abuse and establish core service requirements e.g. accessible facilities and appropriate referral pathways.
	Promote evidence based strategies for improving access to maternity care for disabled women experiencing domestic abuse
	Incorporate outward looking improvement strategies which capitalise on community resources and involve colleagues in community education and health promotion
	Involve other agencies in improving access to maternity services and ensure that non-maternity services promote the importance of early prenatal care
Practice	Foster positive, non-judgmental relationships with disabled women who experience domestic abuse
	Women centered care that does perpetrate negative stereotypes about disabled women
	Develop and implement evidenced based staff education in disability and domestic abuse issues
	Improve access and utilisation of maternity care through multidisciplinary collaboration

## Competing interests

The authors declare that they have no competing interests.

## Authors’ contributions

JPB carried out literature searching, co-ordinated and participated in screening, data extraction, data analysis and drafted the manuscript. CBJ conceived of the study, contributed to screening, data extraction, data analysis and helped to draft the manuscript. JD, TK, AL and JT participated in screening, data extraction, data analysis and provided feedback on drafts of the paper. All authors read and approved the final manuscript.

## Pre-publication history

The pre-publication history for this paper can be accessed here:

http://www.biomedcentral.com/1471-2393/14/234/prepub

## Supplementary Material

Additional file 1 **Search strings used in medline and embase showing the number of results.** This file shows the full search strings used within two of the electronic databases searched. Click here for file

Additional file 2 **Summary of articles included in the review.** This file provides a summary of each of the articles meeting the inclusion criteria and included in the review. Click here for file

Additional file 3 **PRISMA checklist.** This file provides evidence that the systematic review has been conducted and presented in accordance with PRISMA guidelines. Click here for file
