# Supplementary material for: Access and utilisation of maternity care for disabled women who experience domestic abuse: a systematic review
Source: BMC Pregnancy Childbirth. 2014 Jul 17;14:234. doi: 10.1186/1471-2393-14-234 (PMC4223363; doi:10.1186/1471-2393-14-234)
Supplement: Additional file 1 — Search strings used in medline and embase showing the number of results. This file shows the full search strings used within two of the electronic databases searched. [file 1471-2393-14-234-S1.docx]

| 1 | exp Pregnancy/ (1278454) |
| --- | --- |
| 2 | exp Delivery, Obstetric/ (171535) |
| 3 | exp Hospitals, Maternity/ (581882) |
| 4 | exp Prenatal Care/ (124304) |
| 5 | exp Maternal Welfare/ (15097) |
| 6 | exp Obstetrics/ (43092) |
| 7 | exp Maternal Health Services/ (3212805) |
| 8 | exp Cesarean Section/ (89704) |
| 9 | exp Intensive Care, Neonatal/ (21892) |
| 10 | exp Neonatal Nursing/ (5932) |
| 11 | exp Neonatal Screening/ (17455) |
| 12 | exp Intensive Care Units, Neonatal/ (26699) |
| 13 | exp Prenatal Care/ (124304) |
| 14 | exp Prenatal Diagnosis/ (135024) |
| 15 | exp Prenatal Exposure Delayed Effects/ (33559) |
| 16 | exp Postnatal Care/ (73898) |
| 17 | exp Postpartum Period/ (85052) |
| 18 | exp Perinatal Care/ (39445) |
| 19 | exp Midwifery/ (34107) |
| 20 | exp Pregnant Women/ (28930) |
| 21 | exp Family Planning Services/ (54434) |
| 22 | exp Reproductive Health/ (7335) |
| 23 | exp Pregnancy Complications/ (428871) |
| 24 | exp Parturition/ (21757) |
| 25 | exp Doulas/ (62) |
| 26 | 1 or 2 or 3 or 4 or 5 or 6 or 7 or 8 or 9 or 10 or 11 or 12 or 13 or 14 or 15 or 16 or 17 or 18 or 19 or 20 or 21 or 22 or 23 or 24 or 25 (4918437) |
| 27 | (matern$ adj2 (health care or health service$)).mp. [mp=ti, ab, ot, nm, hw, kf, ps, rs, ui, an, sh, tn, dm, mf, dv, kw] (16889) |
| 28 | 1 or 2 or 3 or 4 or 5 or 6 or 7 or 8 or 9 or 10 or 11 or 12 or 13 or 14 or 15 or 16 or 17 or 18 or 19 or 20 or 21 or 22 or 23 or 24 or 25 or 26 or 27 (4918980) |
| 29 | exp Disabled Persons/ (67408) |
| 30 | exp Intellectual Disability/ (404329) |
| 31 | exp disability/ (92750) |
| 32 | *social disability/ (490) |
| 33 | 29 or 30 or 31 or 32 (552735) |
| 34 | (disabled adj1 (person or people or individual or mother or mum)).mp. [mp=ti, ab, ot, nm, hw, kf, ps, rs, ui, sh, tn, dm, mf, dv, kw] (26578) |
| 35 | ((person$ or people) adj2 disab$).mp. [mp=ti, ab, ot, nm, hw, kf, ps, rs, ui, sh, tn, dm, mf, dv, kw] (64185) |
| 36 | ((physical or neurological or sensory or cognitive or mental) adj2 impair$).mp. [mp=ti, ab, ot, nm, hw, kf, ps, rs, ui, sh, tn, dm, mf, dv, kw] (89145) |
| 37 | (Disab$ adj2 (women or woman or female)).mp. [mp=ti, ab, ot, nm, hw, kf, ps, rs, ui, sh, tn, dm, mf, dv, kw] (30922) |
| 38 | 34 or 35 or 36 or 37 (174600) |
| 39 | 33 or 38 (622821) |
| 40 | *"dissent and disputes"/ or exp family conflict/ (10584) |
| 41 | exp Rape/ (11455) |
| 42 | exp Spouse Abuse/ (11361) |
| 43 | exp Battered Women/ (4809) |
| 44 | exp Domestic Violence/ (73323) |
| 45 | exp family violence/ (36173) |
| 46 | exp partner violence/ (5768) |
| 47 | exp emotional abuse/ (213) |
| 48 | exp physical abuse/ (272) |
| 49 | exp sexual abuse/ (45008) |
| 50 | (domestic adj2 (violen$ or abus$ or assault$ or coercion or control)).mp. [mp=ti, ab, ot, nm, hw, kf, ps, rs, ui, sh, tn, dm, mf, dv, kw] (14207) |
| 51 | (marital adj2 (violen$ or abus$ or assault$ or coercion or control)).mp. [mp=ti, ab, ot, nm, hw, kf, ps, rs, ui, sh, tn, dm, mf, dv, kw] (575) |
| 52 | (spouse adj2 (violen$ or abus$ or assault$ or coercion or control)).mp. [mp=ti, ab, ot, nm, hw, kf, ps, rs, ui, sh, tn, dm, mf, dv, kw] (6288) |
| 53 | (partner adj2 (violen$ or abus$ or assault$ or coercion or control)).mp. [mp=ti, ab, ot, nm, hw, kf, ps, rs, ui, sh, tn, dm, mf, dv, kw] (10086) |
| 54 | 40 or 41 or 42 or 43 or 44 or 45 or 46 or 47 or 48 or 49 or 50 or 51 or 52 or 53 (110362) |
| 55 | 28 and 39 and 54 (959) |
