# Supplementary material for: Access and utilisation of maternity care for disabled women who experience domestic abuse: a systematic review
Source: BMC Pregnancy Childbirth. 2014 Jul 17;14:234. doi: 10.1186/1471-2393-14-234 (PMC4223363; doi:10.1186/1471-2393-14-234)
Supplement: Additional file 2 — Summary of articles included in the review. This file provides a summary of each of the articles meeting the inclusion criteria and included in the review. [file 1471-2393-14-234-S2.docx]

| Author, year  [reference no.] | Setting | Design | Objectives | Disability represented | Sample | Key Findings |
| --- | --- | --- | --- | --- | --- | --- |
| Ferri et al. 2007.  [35] | Brazil | Quant | To investigate the independent and interactive effects of domestic violence and mental disorder on neonatal outcomes among pregnant adolescents in a disadvantaged population from Sao Paulo, Brazil | Mental health | Consecutive adolescents (11-19yrs) were invited to participate in interviews 4-48 hours after delivery (n=930) | The effects of violence during pregnancy and the presence of common mental disorder within the last 12 months were additive rather than multiplicative, with no statistical interaction. Pre-term birth was linked to mental disorder but not to violence, whereas small gestational age was linked to violence and not to mental disorder.  The risk of LBW decreased significantly with the number of antenatal consultations (p=0.03) |
| Huth-Bocks et al. 2002.  [40] | USA | Quant | To examine the relationship between prenatal domestic violence and maternal and infant health outcomes | Mental health | 202 women attending community agencies, clinics and social services (68 abused women, 134 non-abused women). | Abused women were significantly more likely to: enter prenatal care later; have significantly longer stay in hospital, higher number of ER visits, and higher number of visits to doctor for the infant. Social support moderated between domestic violence and negative health outcomes.  Domestic violence was strongly associated with maternal depression, but maternal depression is not a mediator between domestic violence and negative health outcomes. |
| Kim et al. 2006.  [41] | USA | Quant | To determine the prevalence of antenatal psychiatric illness in predominantly Spanish and English speaking obstetric patients and examine the association between psychiatric diagnosis during pregnancy and inadequate prenatal care | Mental health | 154 women attending prenatal appointments at a University Hospital in the USA during an 8 month period. | 29% screened positive for psychiatric disorder. Inadequate prenatal care was significantly associated with domestic abuse in the previous year, but not with *current* psychiatric diagnosis, alcohol abuse, primiparity, marital status, receipt of govt. assistance or unplanned pregnancy. |
| Nunes et al. 2010.  [42] | Brazil | Quant | To estimate the prevalence of violence during pregnancy, identify the characteristics associated with abuse and assess the impact on newborn outcomes. | Mental health | 652 women attending pregnant women attending prenatal clinics, with gestational age ranging from 16-36 weeks. | Women experiencing violence had more frequently inadequate number of prenatal visits and insufficient pregnancy weight gain. |
| Lipsky et al. 2004  [43] | USA | Quant | To examine the impact of IPV during pregnancy on antenatal hospitalisation not associated with delivery | Mental health | Police records and hospital case files for women with police reported incidents of IPV who had subsequently had a life birth or fetal death (n=389 incidents). | Logistic regression analysis suggests that exposure to any police reported IPV increases the likelihood of antenatal hospitalisation. |
| Mitra and Manning. 2012.  [36] | USA | Quant | To describe the prevalence of physical abuse in the 12 months before pregnancy and during pregnancy in women with and without disabilities | Unspecified: women self-reported as disabled/non-disabled | Data from the state wide MA-PRAMS survey (Mass. Pregnancy Risk Assessment Monitoring System) in Massachusetts (n= 2,876 of which 138 had a self-reported disability) | Disabled women are significantly more likely to experience physical abuse before during and after pregnancy than women without disabilities.  Prenatal care providers are equally likely to discuss abuse by husbands and partners to women with (54.3%, 95% CI = 42.7-65.5%) and without disabilities (59.4%, 95% CI = 56.8-61.9%). |
| Pandey et al. 2012.  [38] | India | Quant | To understand the effect of women’s empowerment in developing blindness during pregnancy | Visual impairment | Data from the National Family Health Survey of India for women who had given birth in the last 5 years (n=35,248) | 12% experienced blindness during their last pregnancy. Youth, abuse and poor education were associated with blindness.  37% achieved the WHO recommended minimum of 4 prenatal visits. Barriers included: cost; distance; quality. Women’s empowerment was a protective factor against developing blindness during pregnancy but antenatal visits did not reduce the risk of blindness. |
| Webster et al. 1996.  [39] | Australia | Quant | To determine whether pregnancy and neonatal outcomes differ between abused and non-abused women. | Physical impairment | Medical records of 1014 pregnant women attending an Australian hospital were reviewed. Pregnancy outcomes were compared for 242 women reporting past abuse and 59 women reporting abuse during their current pregnancy, and the remaining women not experiencing abuse. | Abuse was associated with: significantly higher use of prescribed drugs and antidepressants; personal history of asthma and epilepsy; smaller support networks; more frequent contact with social work.  Antenatal visits, week’s gestation at first antenatal visit and at delivery were similar for non-abused and abused women. The number of pregnancy admissions was higher among abused women, although this was not statistically significant. |
| Smith et al. 2004.  [45] | Zambia | Qual | To explore how well the safe motherhood and reproductive health services meet the needs of disabled women in Lusaka, Zambia. | Physical impairment | Semi structured interviews with women (n=20) and health practitioners (n=25). 4 women also took part in a preliminary focus group. | Many women disclosed sexual exploitation and not having a stable partner. Barriers to services include: physical accessibility of facilities; poverty; costly and inaccessible transport; negative attitudes of practitioners are other non-disabled patients. |
| Nosek et al. 2001  [37] | USA | Mixed | To explore the psychosocial behaviours of women with physical disabilities | Physical impairment | Qualitative interviews with 31 women with physical impairments.  946 questionnaires (475 completed by disabled women, 406 non-disabled women) | 56% disabled women reported services had difficulty accommodating their birth. Barriers to reproductive healthcare included: practitioners unwilling to assist in high risk pregnancies; poor communication with practitioners; lack of knowledge about disability issues; negative attitudes about disability. |
| Kopac and Fritz. 2004  [44] | USA | Mixed | To examine the accessibility of gynaecological and reproductive services for women with developmental disabilities from the perspective or practice nurses | Developmental disabilities | Questionnaires mailed to 3451 nurses via professional membership organisations across the USA (n=727) | 81.4% respondents identified that there were barriers to care for women with developmental disabilities. Barriers included: nurses’ lack of empathy, knowledge and patience; women’s fear of examinations; difficulties communicating and giving consent; services not provided through social insurance; inaccessible offices and equipment; negative attitudes about disability. |
